# The Bacterial Population of Neutral Mine Drainage Water of Elizabeth’s Shaft (Slovinky, Slovakia)

**DOI:** 10.1007/s00284-018-1472-6

**Published:** 2018-03-12

**Authors:** Jana Kisková, Zuzana Perháčová, Ladislav Vlčko, Jana Sedláková, Simona Kvasnová, Peter Pristaš

**Affiliations:** 1 Institute of Biology and Ecology, Faculty of Science, Pavol Jozef Šafárik University in Košice, 041 54 Košice, Slovakia; 2 Department of Biology and General Ecology, Faculty of Ecology and Environmental Sciences, Technical University in Zvolen, 960 53 Zvolen, Slovakia; 3 Department of Biology and Ecology, Faculty of Natural Science, Matej Bel University, 974 01 Banská Bystrica, Slovakia; 4 Institute of Animal Physiology, Centre of Biosciences of Slovak Academy of Sciences, 041 01 Košice, Slovakia

## Abstract

Although neutral mine drainage is the less frequent subject of the interest than acid mine drainage, it can have adverse environmental effects caused mainly by precipitation of dissolved Fe. The aim of the study was to characterize the composition of bacterial population in environment with high concentration of iron and sulfur compounds represented by neutral mine drainage water of Elizabeth’s shaft, Slovinky (Slovakia). Direct microscopic observations, cultivation methods, and 454 pyrosequencing of the 16S rRNA gene amplicons were used to examine the bacterial population. Microscopic observations identified iron–oxidizing Proteobacteria of the genera *Gallionella* and *Leptothrix* which occurrence was not changed during the years 2008–2014. Using 454 pyrosequencing, there were identified members of 204 bacterial genera that belonged to 25 phyla. Proteobacteria (69.55%), followed by Chloroflexi (10.31%) and Actinobacteria (4.24%) dominated the bacterial community. Genera *Azotobacter* (24.52%) and *Pseudomonas* (14.15%), followed by iron–oxidizing Proteobacteria *Dechloromonas* (11%) and *Methyloversatilis* (8.53%) were most abundant within bacterial community. Typical sulfur bacteria were detected with lower frequency, e.g., Desulfobacteraceae (0.25%), Desulfovibrionaceae (0.16%), or Desulfobulbaceae (0.11%). Our data indicate that the composition of bacterial community of the Elizabeth’s shaft drainage water reflects observed neutral pH, high level of iron and sulfur ions in this aquatic habitat.

**Electronic supplementary material:**

The online version of this article (10.1007/s00284-018-1472-6) contains supplementary material, which is available to authorized users.

## Introduction

Studies of the diversity of microorganisms inhabiting extreme environments have increased significantly over the past years. These environments were far more widespread during the early life of our planet and organisms isolated from these sites are representative of archaic life forms. Extremophiles as living organisms or as sources of enzymes and other cell products offer a wide range of applications in a variety of industrial and biotechnological operations also in medicine [1].

Mining activities and ore processing result in irreversible changes in landscape in the form of heaps and sewage sludge beds of waste material. These deposits are permanent source of toxic substances, especially heavy metals which contaminate all environmental compounds, mainly soil and water. Changes of water and soil quality affect also biodiversity of mining area [2–5]. The properties of drainage water depend on many factors, including mineralogical, geochemical properties, hydrogeological conditions, and the activity of lithoautotrophic microorganisms. Oxidative dissolution of sulfide minerals generates acidity and releases sulfate, iron, and associated metals to pore waters. This phenomenon is known as acid mine drainage (AMD). However, neutral mine drainage (NMD) conditions may persist in an abundance of carbonate minerals [6–9]. Principal threats to water quality under circumneutral pH conditions are weakly hydrolysing metals, including Fe, Ni, Cu, and Zn [10]. While mechanisms of sulfide mineral oxidation in AMD and bacteria participating in are well established, there are limited data on microbiota of NMD settings.

Slovinky mining area (north part of Slovak Ore Mountains territory, Spišská Nová Ves district, Slovakia) is considered the largest source of copper ores in Slovak region (chalcopyrite, cuprite, malachite, delafossite) but it is also rich in iron ores (siderite, pyrite, chalcopyrite, delafossite). The most frequent secondary and tertiary minerals are iron oxides such as goethite or crystalline hydrous ferric oxide [11]. Mining in Slovinky was stopped in 1999 but due to sludge bed vulnerability represent high-risk area threatening all environmental components (soil, water, living organisms). The mine dumps are near-neutral or slightly alkaline (pH 7.2–8.8) because the acidity generated by the decomposition of the sulfide ores is efficiently neutralized by the abundant carbonate minerals [2, 11]. Elizabeth’s shaft was built in 1900 and it works as mining drainage water system. Sulfide minerals exposed by mining and erosion are unstable in the presence of atmospheric oxygen and water; the resulting oxidation of sulfides can release sulfate and iron ions into the drainage water [8]. Regular monitoring of physico-chemicals parameters of mine drainage water demonstrated that an average iron concentration did not exceed 0.5 mg/l for many years. In addition, a high concentration of SO_4_, Mn, As, and Sb have been long-term monitored [12]. Based on these characteristics, we assumed a high incidence of iron and sulfur bacteria within bacterial community in drainage water.

Iron bacteria represent heterogeneous group belonging to many different phyla (e.g., Proteobacteria, Firmicutes, Nitrospirae) which can be divided into several groups based on their physiological properties and the role in ferrous ion oxidation [13]. Originally, iron bacteria were considered to catalyze the oxidation of iron II (Fe^2+^, ferrous iron) to iron III (Fe^3+^, ferric iron). More recently, bacteria catalyzing the reduction of ferric to ferrous ion have been also included in iron bacteria [14]. *Gallionella ferruginea* is probably the oldest known iron-oxidizing bacterium [15]. The bacterium was first discovered in the ochre mineral deposit and to date *Gallionella*-related species were found in many various soil and aquatic habitats, always associated with iron. *Gallionella* spp. is one of the most commonly detected bacteria in acid mine environments [16]. *Leptothrix* spp. are heterotrophic Fe/Mn-oxidizing inhabitant of aqueous environments, especially characterized by a circumneutral pH to slightly acidic, an oxygen gradient and a source of reduced Fe and Mn minerals [17]. Both of iron-oxidizing bacterial genera are characterized by biogenically formed various iron oxyhydroxide structures. *Gallionella* spp. are characterized by stalk and particulates formation; *Leptothrix* spp. by typical sheaths easily recognized by light microscopy [14, 18–21].

Sulfur bacteria represent a diverse group of microorganisms capable of metabolizing sulfur and its compounds [22]. However, in most environments (particularly in the sub-surface) few bacterial species, or combination of species, probably carry out iron and organic carbon oxidation, carbon and nitrogen fixation, extracellular polymeric slime production, as well as iron and sulfur reduction leading to the either to the AMD or NMD. Members of the genus *Acidithiobacillus* (particularly A. *ferrooxidans*) are frequently found in acid mine environments with high occurrence of iron-sulfur ores but less often under circumneutral to alkaline conditions. These bacteria oxidize sulfide minerals, resulting in ferric ions and sulfuric acid production and acidification of the environment [23, 24].

The original aim of this study was to examine the occurrence of iron-sulfur bacteria in a neutral mine drainage of Elizabeth’s shaft using direct microscopy and cultivation methods and to investigate their seasonal dynamics over several years. Since some changes in pH of drainage water have been recorded during the period of investigation, we decided to analyze the structure of bacterial community in NMD using a high-throughput sequencing technique in order to detect bacteria that could contribute to (or responsible for) these changes.

## Materials and Methods

### Sampling Site and Physico-Chemical Water Quality Indicators Measurements

Mine drainage water samples were collected directly from the water flowing out from Elizabeth’s shaft in Slovinky village, Slovakia (48°52′43″N 20°50′38″E) (Fig. S1). Five-hundred microliters of water was taken two times a year in the period from 2008 to 2011 (first week in March and October), and four times a year in the years 2012–2014 (first week in March, June, August, and October) into sterile bottles. The water was transported under cold, dark conditions to the laboratory. Physico-chemical parameters (pH and electric conductivity) were measured directly in the field with WTW Multi 340i instrument (WTW Gmbh, Weilheim, Germany) equipped with a pH electrode WTW Sen Tix 31-3 and standard WTW TetraCon 325 electrode for an electric conductivity (total dissolved solids, TDS) measurement.

### Direct Microscopic Observations and Cultivation-Based Analyses

The light microscope Olympus BX 40 equipped with digital camera was used for quantitative and qualitative assessment of abioseston and bioseston in mine drainage water samples.

First, 10 ml water from each sample was taken into 50-ml centrifuge tube with conical bottom and centrifuged at 2000 g for 5 min. Subsequently, supernatant was removed and sample was spin down briefly to remove drops from the inside of the tube. A small water drop was taken by Pasteur pipette, placed into Cyrus chamber and examined by light microscopy. Bacteria determination was performed according to Švorcová’s [25] and Tóthová and Mogoňová’s [26] instructions. Relative abundance of abioseston and bioseston fraction was expressed by the percentage of coverage of the ten different microscopic fields at ×1000 total magnifications [26].

The selective medium proposed by Švorcová [25] was used to detect subpopulation of selected iron bacteria ((NH_4_)_2_SO_4_, NaNO_3_, K_2_HPO_4_·3H_2_O, and MgSO_4_·7H_2_O each of 0.5 g, 3 g of FeSO_4_, 10 g sodium citrate, 2 g of sucrose, 1 g tryptose, 20 g agarose, distilled water add into total volume of 1 l, pH 6.8). Components were dissolved in distilled water and sterilized at 100 °C for 30 min by fractional sterilization (three consecutive days in flowing water). One hundred microliters of drainage water was spread directly onto solid medium and cultivated at 25 °C for 3 days. Obtained bacterial colonies were picked up by a sterile microbial loop, stained using Gram’s method and examined by light microscopy.

To detect the presence of iron–sulfur oxidizing bacteria of the genus *Acidithiobacillus*, the water samples (1 ml) were inoculated into 50 ml of liquid Thiobacillus Broth (HiMedia, Mumbai, India) and cultivated aerobically at 25 °C until turbidity developed. Serial dilution of the culture was then spread on the solid Thiobacillus Agar (HiMedia, Mumbai, India) and cultivated at 25 °C for 3 days. Also cultivation of water samples was carried out at 30 °C for 3–4 days in a standard 9 K medium [27].

Non-parametrical Kruskal–Wallis test (one-way ANOVA) was used to analyze the changes in physico-chemical parameters and occurrence of iron bacteria and/or other microorganisms detected by microscopic observation during the period of investigation.

### DNA Isolation, Bacterial 16S rDNA PCR Amplification and 454 Pyrosequencing

In March 2014, a high-throughput sequencing analysis of 16S rRNA gene was performed in the order to better understand the composition of bacterial community in drainage water of Elizabeth’s shaft.

One hundred millilitres of the drainage water was centrifuged at 3000 g for 20 min and the total metagenomic DNA was extracted from the pellet using the GenElute™ Bacterial Genomic DNA Kit (Sigma-Aldrich, St. Louis, USA). The quality of DNA was checked by agarose gel electrophoresis.

The bacterial barcoding was performed using universal primers (fwd: 5′-TAGAGTTTGATYMTGGCTCAG-3′ and rev: 5′-GWATTACCGCGGCKGCTG-3′) to amplify an approximately 500 bp fragment consisting of the hypervariable V1–V3 region of the 16S rRNA gene [28, 29]. Primers were modified by the addition of a GS FLX Titanium Series adapter sequences A and B (A: CCATCTCATCCCTGCGTGTCTCCGAC and B: CCTATCCCCTGTGTGCCTTGGCAGTC) and four-base library “key” sequence (TCAG). Multiplex identifier (MID) sequence specifying this sample was incorporated into the forward primer (ACTATACGAG).

PCR reaction mixture consisted of 0.1 µl of Phire® Hot Start II DNA Polymerase (Finnzymes Oy, Espoo, Finland), 0,2 mM dNTPs (Metabion, Martinsried, Germany), 1 × Phire Reaction Buffer, 10 pmol of each primer and 40–80 ng of DNA template in a final volume of 20 µl. The PCR conditions consisted of initial denaturation step of 95 °C for 3 min followed by 35 cycles of 93 °C for 60 s, 50 °C for 60 s, and 72 °C for 70 and a final extension step of 72 °C for 5 min (C1000 Thermal Cycler, Bio-Rad Laboratories GmbH, München, Germany). PCR product was checked for correct size on a 1% agarose gel, purified with Wizard® SV Gel and PCR Clean-Up System (Promega, Madison, USA). The next generation 454 pyrosequencing was performed using Roche 454 GS-FLX Titanium instrument (LaRoche, USA) and reagents according to the manufacturer’s guidelines. Demultiplexed raw reads generated in this study have been deposited in the NCBI Sequence Read Archive with the Bioproject accession number PRJNA324333.

### Processing of 454 Data

DNA sequencing data were processed using the Mothur bioinformatics software package (version 1.36.1) [30].

First, flow gram file was produced from the standard flow gram file (sff) using “sffinfo” command. The resulting flow gram file was trimmed using “trim.flows.” Flow grams differed > 2 bases from primer sequence and > 1 base from barcode were discarded. The flow gram file was denoised using “shhh.flows” as Mothur’s implementation of PyroNoise algorithm [31]. After denoising, adapters, barcodes, and primers were trimmed from sequences (“trim.seqs”). Sequences of low quality (average quality score < 0.25), shorter than 200 bp, containing ambiguously determined nucleotides and homopolymer (maximum = 8) with > 2 base-difference from primer and > 1 base-difference from barcode were excluded from further analysis. Trimmed sequences were aligned (align.seqs) against the Silva database (silva.seed_v123) [32]. Chimeric sequences were filtered out using Uchime algorithm implemented in Mothur (chimera.uchime) [33]. Obtained high-quality non-chimeras sequences were processed using Mothur’s pipeline (unique.seqs, remove.lineage, filter.seqs, unique.seqs, dist.seqs) and after that they were clustered into operational taxonomic units (OTUs) with a 97% similarity threshold (“cluster” command) using the average neighbor method. OTUs represented by a single sequence were excluded from the following analysis. Representative sequence of each OTU, produced using “get.oturep” command, was assigned by RDP Classifier at the confidence level of > 0.80 (http://rdp.cme.msu.edu) [34].

## Results

### Physico-Chemical Parameters and Direct Microscopic Observations

**Fig. 1:**
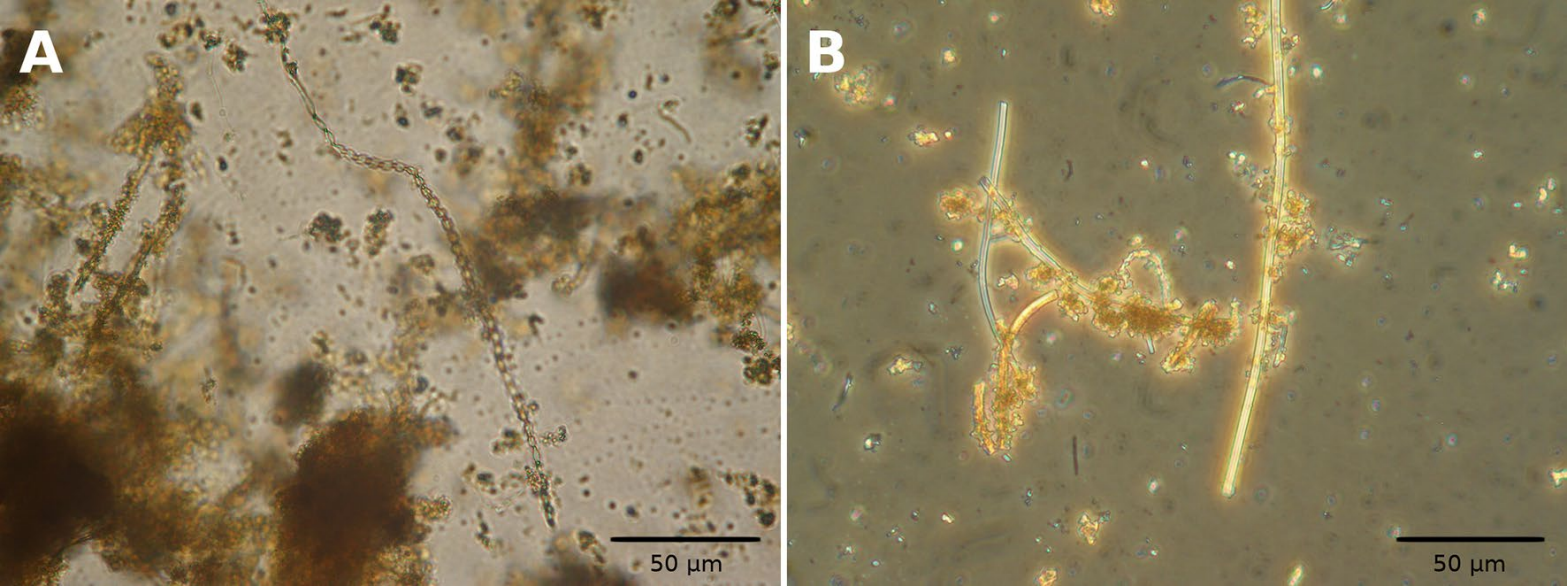
*Gallionella* spp. (**a**) and *Leptothrix* spp. (**b)** observed in mine drainage water of Elizabeth’s shaft (Slovinky, Slovakia). Microscopic observation at ×1000 total magnification

Direct microscopic observations of water samples showed that the abioseston (percentage of coverage ranged from 8.0 to 10%) was dominated by iron and manganese clots with occasional occurrence of dead plant tissues parts and other organic residues (such as pollen, insect wings etc.). Diatoms of genera *Nitzschia, Navicula*, and *Eunotia* were dominant group of bioseston (percentage of coverage ranged from 2.5 to 3.5%). Iron bacteria in the drainage waters of Elizabeth’s shaft were represented by a relatively small number (percentage of coverage ranged from 1.0 to 2%) (Table 1). One group of iron bacteria was determined as of *Gallionella* spp. forming stalks and particules characteristic for *Gallionella ferruginea*. Another group of iron bacteria was determined as *Leptothrix* spp. based on the presence of a dominant sheath typical for *Leptothrix ochracea* (Fig. 1).

**Table 1:** Physico-chemicals parameters and microscopic observation of abioseston and bioseston of mine drainage water from Elizabeth’s shaft measured in the years 2008–2014 (Slovinky, Slovakia)

Years	2008 (*n* = 2)	2009 (*n* = 2)	2010 (*n* = 2)	2011 (*n* = 2)	2012 (*n* = 4)	2013 (*n* = 4)	2014 (*n* = 4)	*P* value
pH^a^	7.1 ± 0.3	7 ± 0.1	6.9 ± 0.1	6.9 ± 0.3	6.8 ± 0.3	6.7 ± 0.2	6.5 ± 0.2	> 0.05
EC [mS/m]^a^	42 ± 1.4	50 ± 1.4	49 ± 4.2	54 ± 2.8	57.3 ± 2.3	61.5 ± 1.3	63.5 ± 1.3	< 0.001
TDS [mg/l]^a^	172 ± 2.8	181 ± 1.4	185 ± 2.8	198 ± 4.2	201.5 ± 3.1	200.3 ± 1.7	220.3 ± 2.6	< 0.001
Abioseston^b^	10 ± 1.4	9.5 ± 0.7	9.5 ± 0.7	9.5 ± 0.7	8.5 ± 2.4	8.0 ± 1.8	8.5 ± 1.7	> 0.05
Diatoms^b^	3.5 ± 0.7	3.5 ± 0.7	3 ± 0.0	4.0 ± 1.4	3 ± 1.2	2.5 ± 1.3	3 ± 0.8	> 0.05
Gallionella spp.^b^	1 ± 0.0	2 ± 0.0	1 ± 0.0	1 ± 0.0	1.5 ± 0.7	1 ± 0.0	1 ± 0.0	> 0.05
Leptothrix spp.^b^	1 ± 0.0	2 ± 0.0	2 ± 0.0	2 ± 0.0	1 ± 0.0	1.5 ± 0.7	1 ± 0.0	> 0.05

*EC* electric conductivity, *TDS* total dissolved solids, *P* value result of Kruskal–Wallis test (one-way ANOVA)

^a^Values are expressed as arithmetic mean value ± standard deviation of measurements in a given year (*n*)

^b^Values are expressed as arithmetic mean value ± standard deviation of the percentage of coverage of 10 different microscopic fields measurements in a given year (*n*)

Cultivation analysis on the selective medium according to Švorcová [25] confirmed the presence of *Ferribacterium* species in each sampling during the period of investigation. Bacterial colonies were rusty with a white border or rust-shiny gold. Gram staining and microscopic observation shown the presence of small gram-negative rod-shaped, elliptical cells living in pairs or chains with surface coated by gelatinous pouch or thin capsule. These characteristics are typical for the bacteria of *Ferribacterium* genus [26, 35]. Cultivation using the selective Thiobacillus agar and 9K medium did not evidence the presence of bacteria belonging to *Acidithiobacillus* spp. in any sample.

During the years 2008–2014, the salinity (TDS) of Elizabeth’s shaft drainage water significantly increased from 172 to 220.25 mg/l (pH < 0.001) and pH value slightly degreased from 7.1 to 6.5 (*P* > 0.05) but these changes did not affect the incidence of observed bacteria, resp. diatoms (Table 1).

### Bacterial Communities Recovered by 454 Pyrosequencing

**Fig. 2:**
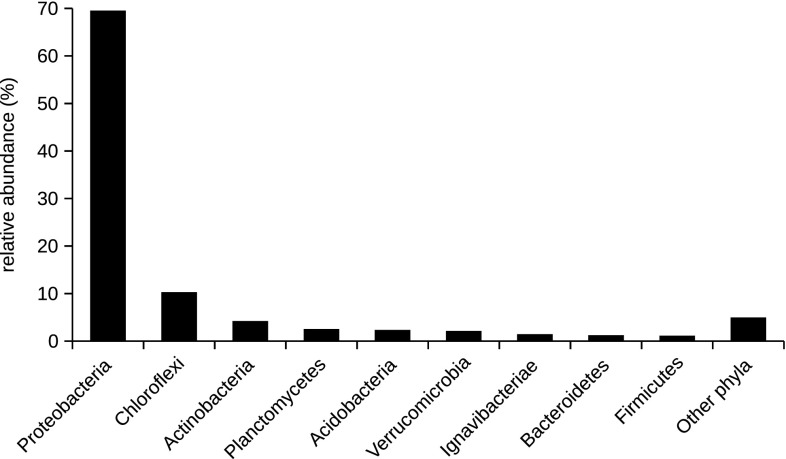
Bacterial composition at the phylogenetic phylum level of mine drainage water of Elizabeth’s shaft (Slovinky, Slovakia). The category “Other phyla” groups bacterial phyla whose relative abundance was below 1%

After trimming, denoising, quality, and length filtering and removing chimeras, a total of 7095 high-quality sequences were obtained. Sequences were clustered into 813 OTUs, while 414 were non-singletons (Table S1). One OTU was classified as Archaea, phylum Aigarchaeota (represented by two sequences). Other sequences were classified into 22 known bacterial phyla and three candidate phyla. The majority of sequences belonged to Proteobacteria (69.55%) followed by Chloroflexi (10.31%), Actinobacteria (4.24%), Planctomycetes (2.57%), Acidobacteria (2.35%), and Verrucomicrobia (2.14%). Other phyla were represented by less than 2% sequences (Fig. 2).

**Fig. 3:**
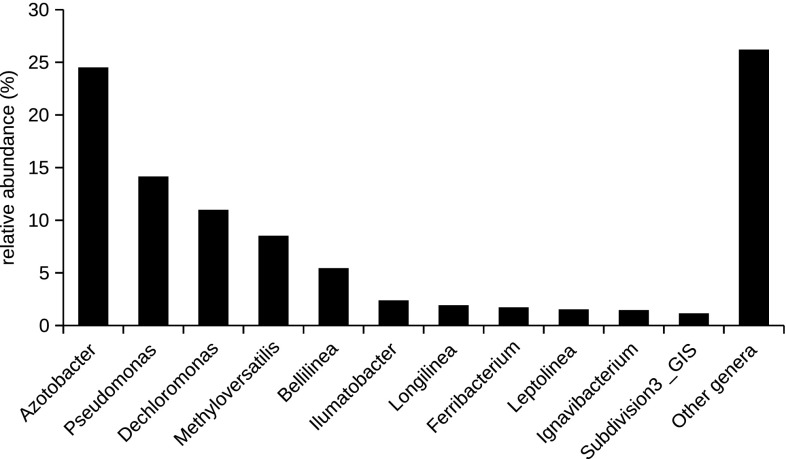
Bacterial composition at the phylogenetic genus level of mine drainage water of Elizabeth’s shaft (Slovinky, Slovakia). The category “Other genera” groups bacterial genera whose relative abundance was below 1%

Bacteria were divided into three groups according to RDP classification (Table S2). The first group included 175 genera with known taxonomy (6645 sequences, 329 OTUs). The second group was represented by 394 sequences (75 OTUs) successfully assigned to known phyla but with unknown classification into other taxonomic levels (such as class, order, family). The third group included 54 sequences (9 OTUs) classified into three candidate phyla. Genus *Azotobacter* was most abundant genus (24.52%) followed by *Pseudomonas* (14.15%), *Dechloromonas* (11%), *Methyloversatilis* (8.53%), and *Bellilinea* (5.46%). Other genera were represented by less than 5% of sequences (Fig. 3).

We detected numerous members of iron and sulfur bacteria group (Table 2). Proteobacteria such as *Dechloromonas* spp. (11%) and *Ferribacterium* spp. (1.73%) dominated within iron bacteria. Other most abundant iron bacteria were Chloroflexi such as *Bellilinea* spp. (5.46%), *Longilinea* spp. (1.93%), and *Leptolinea* spp. (1.54%). Sulfur bacteria were represented by members of families Desulfobacteraceae (0.25%), Desulfovibrionaceae (0.16%), or Desulfobulbaceae (0.11%) or the genera such as *Acidiferrobacter* (0.24%, Ectothiorhodospiraceae), *Sulfuritalea* (0.21%, Rhodocyclaceae), *Thiofaba* (0.18%, Halothiobacillaceae) or *Thiococcus* (0.17%, Chromatiaceae). In addition, *Zoogloea* spp. as very important bacterium from the family Rhodocyclaceae (0.42%) was detected (Table 2).

**Table 2:** Representatives of iron and sulfur bacteria in mine drainage water of Elizabeth’s shaft (Slovinky, Slovakia)

Phylum	Taxonomic affiliation	Nr. of reads	Relative abundance %
Actinobacteria	*Rhodococcus* spp.	30	0.42
Chloroflexi	*Bellilinea* spp.	387	5.46
Chloroflexi	*Leptolinea* spp.	109	1.54
Chloroflexi	*Longilinea* spp.	137	1.93
Proteobacteria	*Acidiferrobacter* spp.	17	0.24
Proteobacteria	*Bilophila* spp.	11	0.16
Proteobacteria	*Desulfatiferula* spp.	8	0.11
Proteobacteria	*Desulfospira* spp.	7	0.10
Proteobacteria	*Dechloromonas* spp.	780	11.00
Proteobacteria	*Ferribacterium* spp.	123	1.73
Proteobacteria	*Leptothrix* spp.	13	0.18
Proteobacteria	*Geobacter* spp.	9	0.13
Proteobacteria	*Geopsychrobacter* spp.	8	0.11
Proteobacteria	*Sulfuritalea* spp.	15	0.21
Proteobacteria	*Thiobacter* spp.	6	0.08
Proteobacteria	*Thiococcus* spp.	12	0.17
Proteobacteria	*Thiofaba* spp.	13	0.18
Proteobacteria	*Thiothrix* spp.	6	0.08

Complete bacterial classification according to RDP database (http://rdp.cme.msu.edu) is shown as a supplementary material in ESM3.xlsx file.

## Discussion

One of the most critical issues in mine environments is the natural oxidation (chemical and biological) of sulfide mineral tailings that are exposed to water, oxygen, and microorganisms. This oxidation is responsible for the generation of mine drainage that compromises the quality of soil, surface water, and sub-surface water bodies, hence affecting overall biodiversity [2–5].

Despite expectations, direct microscopic observations and the high-throughput analysis have shown a low abundance of *Gallionella* spp. and *Leptothrix* spp. Even *Gallionella* spp. was not detected by 454 pyrosequencing. The low occurrence of these bacteria may be due to preference for more acidic conditions as well as other environmental factors (e.g., heavy metals).

*Ferribacterium* is a genus of the family Rhodocyclaceae and up to now, only one species is known (*F. limneticum*). This bacterium belongs to Fe(III)-reducing bacteria and was first isolated from mining-impacted fresh lake sediments containing heavy metals such as Fe, Pb, Zn, or As [36]. Its presence in drainage water of Elizabeth’s shaft was confirmed by cultivation as well as 454 pyrosequencing.

*Acidithiobacillus* species was detected neither by cultivation methods nor by 454 pyrosequencing. These findings confirmed the preference of the genus *Acidithiobacillus* of acidic environment, despite the high sulfur and iron content in neutral mine drainage water.

The chemistry of mine drainage is the result of the competing processes of acid formation and neutralization [37]. Several mine discharges are characterized by a circumneutral pH due to either an absence of pyrite within the ore, hence minimizing the acid generating potential on site, or due to the presence of a carbonate minerals as a calcite, which effectively neutralize any acidity produced [11]. Drainage water flowing from Elizabeth’s shaft is characterized by high concentration of iron, manganese, arsenic, and sulfate ions [12]. Concentration of copper and zinc did not overexpress limit values according of Regulation of the Government of the Slovak Republic [38]. Continual increase of TDS concentration is probably due to the gradual release of metal(loid)s during sulfide mineral oxidation and bacterial metabolic activity. The Elizabeth’s shaft drainage water could be defined as circumneutral, since acid generated via sulfide mineral oxidation (e.g., pyrite or chalcopyrite) is neutralized by the dissolution of carbonate minerals as siderite or malachite. On the other side, our results indicate a slight shift to the acidic pH during the examined period. Slow reduction of pH of drainage water is supported by earlier studies demonstrating pH value of 8.24 in 1999 and 7.56 in 2000 [39].

Therefore, we performed an analysis of the bacterial community using a high-throughput sequencing technique to find out bacteria that could be involved in reducing pH of drainage water. Generally, Proteobacteria, Nitrospirae, Actinobacteria, and Firmicutes are most frequently detected phyla in AMD [6, 24, 40]. Proteobacteria, Deinococcus/Thermus, Gemmatimonadetes, Acidobacteria, and Actinobacteria were found with high frequency also in neutral mine drainage [3, 41]. While lithotrophic genera such as *Acidithiobacillus, Acidiphilum, Ferrovum, Leptospirillum, Gallionella*, and *Sulfobacillus* dominate AMD environments [6, 24, 40], heterotrophic Proteobacteria such as *Pseudomonas* spp., *Bacillus* spp., and *Stenotrophomonas* spp. were found with high abundance in neutral copper mine drainage [42] and other mining samples [43, 44]. The high abundance of *Pseudomonas* spp. was also confirmed in our study. Members belonging to the genus *Pseudomonas* are characterized by great deal of metabolic diversity and they are able to colonize a wide range of environments. A number of studies have been demonstrated its resistance to heavy metals and its capability to degrade a wide range of pollutants [45, 46].

Pereira et al. [3] assumed that the abundance of tolerant bacteria in areas of extreme environmental conditions increases, while that of more sensitive microorganisms decreases. Previous studies have reported that AMD is accompanied by low bacterial diversity [6, 47]. In contrast, a wide range of different bacteria including iron-oxidizing and heterotrophic organisms were found in slightly alkaline French mine sediments [48] and alkaline river sediments contaminated with heavy metals released from Brazilian arsenic mine [49]. Analysis of microbial composition of wastewater of Elizabeth’s shaft showed a trend to increase the abundance of tolerant bacteria leading to a reduction of the total bacterial diversity. Proteobacteria represent almost 70% of the total diversity of bacterial community. These bacteria have been found to be predominant phylum in many mine environments indicating the high adaptability of members to extreme mining environments [3, 48, 49].

Free-living motile bacteria of the genus *Azotobacter* dominate bacterial community in wastewater of Elizabeth’s shaft. These bacteria were found mainly in neutral soil and aquatic environments and they are capable of atmospheric nitrogen fixation due to iron requiring enzymatic system and can survive in contaminated environments by heavy metals. Thus, isolates resistant to heavy metals could be employed in bioremediation processes [50–52].

Interestingly, genus *Methyloversatilis* (Nitrosomonadales, Sterolibacteriaceae) showed relatively high abundance (8.53%) within bacterial population in this study. Recently, only few species belonging to this genus have been found in natural and human-made ecosystems [53–56]. Genus was detected with low frequency in alkaline mountaintop mine drainage in Central Appalachian streams [57].

Rhodocyclales, Rhizobiales, Rhodobacterales, and Rhodospirillales formed relatively large group within Proteobacteria. Many members of these taxa exhibit very versatile metabolic capabilities allowing them survive under various extreme environmental conditions [22]. They were found with high frequency in neutral mine drainage [41], in slightly alkaline mine sediments [48] as well as in AMD [24]. In this study, the highest number of sequences was affiliated to the genus *Dechloromonas*. These bacteria are known as nitrate-dependent neutrophilic iron-oxidizers and perchlorate reducer [58]. Bacteria belonging to this genus were found in soil high concentration of iron also in circumneutral or slightly acidic mine waters contaminated by many different heavy metals [59, 60]. Bacteria of the genus *Zoogloea*, member of the family Rhodocyclaceae, were known as activated sludge bacteria responsible for flock formation in activated sludge and are used in waste water purification processes [61].

Members of the phylum Chloroflexi are frequently detected in polluted environments [62, 63]. The phylum with the predominant family Anaerolineaceae represents about 10% diversity of bacterial community in this study. Similarly, phylum showed a high abundance in alkaline river sediments contaminated with heavy metals [49] and in neutral copper mine drainage [3], but was not detected in AMD [24].

While sulfate-reducing bacteria have been commonly identified in tailings deposits and sulfide mine wastes [7, 64], representatives of this bacterial group (Desulfobacterales, Desulfovibrionales, Desulfuromonadales, and Syntrophobacterales) were detected with relative low frequency in this study. In addition, typical sulfur-oxidizing bacteria (e.g., *Acidiferrobacter* spp. *Thiofaba* spp., *Thiococcus* spp.) were found with low abundance.

Although high-throughput sequencing techniques offer an efficient way to access the microbial community in different environments, several studies have been also demonstrated that bacteria represented rare taxa within bacterial community could not be detected at the sequencing depth [65–67]. Similarly, we did not detected *Gallionella* spp. by 454 pyrosequencing. Moreover, culture-independent methods bring not important information about bacterial pathogenicity, antimicrobial resistance or production of metabolites and enzymes [66]. In practice, only cultivable microorganisms can be effectively used in industrial and biotechnological operations (e.g., bioremediation processes). Therefore, combination of culture-independent and culture-dependent methods is important in bacterial community investigations.

In addition to bacteria, genera of diatoms *Nitzschia* spp., *Navicula* spp. and *Eunotia* spp. were observed with high abundance in drainage water. Diatoms, as one of the dominant components of phytoplankton, are widely distributed in freshwater and marine ecosystems. They respond quickly to environmental changes therefore are popular tool for monitoring water quality. Many studies on metal polluted aquatic ecosystems have shown that diatoms respond through shifts in dominant taxa also at the individual level with changes in frustule morphology [68–70]. Several species belonging to the genera *Nitzschia* spp., *Navicula* spp. and *Eunotia* spp. were detected as dominant or with high abundance within bacterial communities in water samples from mining areas. These areas, similarly to Slovinky mining area, are characterized by the high occurrence of various iron and copper ores and high concentration of Fe, Cu, Pb, Cr, Zn, and other heavy metals in flowing rivers [69, 70].

In conclusion, relatively low abundance of typical iron- and sulfur-bacteria in microbial community indicates that in addition to high concentration of iron and sulfur, other environmental factors significantly affect the composition of bacterial community and bacterial species with a great metabolic diversity dominate among bacteria. The pH of the drainage water is still nearly neutral, however we detected slightly shift to acidic. Sulfide mineral oxidation and possibly metabolic activity of iron/sulfur oxidizers could lead to the continual decrease of pH and to the deterioration of environmental impact of mine drainage of Elizabeth’s shaft. Monitoring of the pH value continues and any changes in the bacterial community will be verified by further metagenomic analysis.

## Electronic supplementary material

Below is the link to the electronic supplementary material.

Supplementary material 1 (PDF 3921 KB)

Supplementary material 2 (PDF 213 KB)

Supplementary material 3 (XLSX 31 KB)
