# Correction to: The Bacterial Population of Neutral Mine Drainage Water of Elizabeth’s Shaft (Slovinky, Slovakia)

**DOI:** 10.1007/s00284-020-01954-z

**Published:** 2020-04-15

**Authors:** Jana Kisková, Zuzana Perháčová, Ladislav Vlčko, Jana Sedláková, Simona Kvasnová, Peter Pristaš

**Affiliations:** 1 Institute of Biology and Ecology, Faculty of Science, Pavol Jozef Šafárik University in Košice, 041 54 Košice, Slovakia; 2 Department of Biology and General Ecology, Faculty of Ecology and Environmental Sciences, Technical University in Zvolen, 960 53 Zvolen, Slovakia; 3 Department of Biology and Ecology, Faculty of Natural Science, Matej Bel University, 974 01 Banská Bystrica, Slovakia; 4 Institute of Animal Physiology, Centre of Biosciences of Slovak Academy of Sciences, 041 01 Košice, Slovakia

## Correction to: Current Microbiology (2018) 75:988–996 https://doi.org/10.1007/s00284-018-1472-6

The article “The Bacterial Population of Neutral Mine Drainage Water of Elizabeth's Shaft (Slovinky, Slovakia)”, written by Jana Kisková, Zuzana Perháèová, Ladislav Vlèko, Jana Sedláková, Simona Kvasnová and Peter Pristaš, was originally published electronically on the publisher’s internet portal on 12 March 2018 without open access. With the author(s)’ decision to opt for Open Choice the copyright of the article changed on 9 April 2020 to © The Author(s) 2018 and the article is forthwith distributed under a Creative Commons Attribution 4.0 International License (https://creativecommons.org/licenses/by/4.0/), which permits use, sharing, adaptation, distribution and reproduction in any medium or format, as long as you give appropriate credit to the original author(s) and the source, provide a link to the Creative Commons licence, and indicate if changes were made. The original article has been corrected.

