# Supplementary figures and images for: The Bacterial Population of Neutral Mine Drainage Water of Elizabeth’s Shaft (Slovinky, Slovakia)
Source: Curr Microbiol. 2018 Mar 12;75(8):988–96. doi: 10.1007/s00284-018-1472-6 (PMC7160218; doi:10.1007/s00284-018-1472-6)

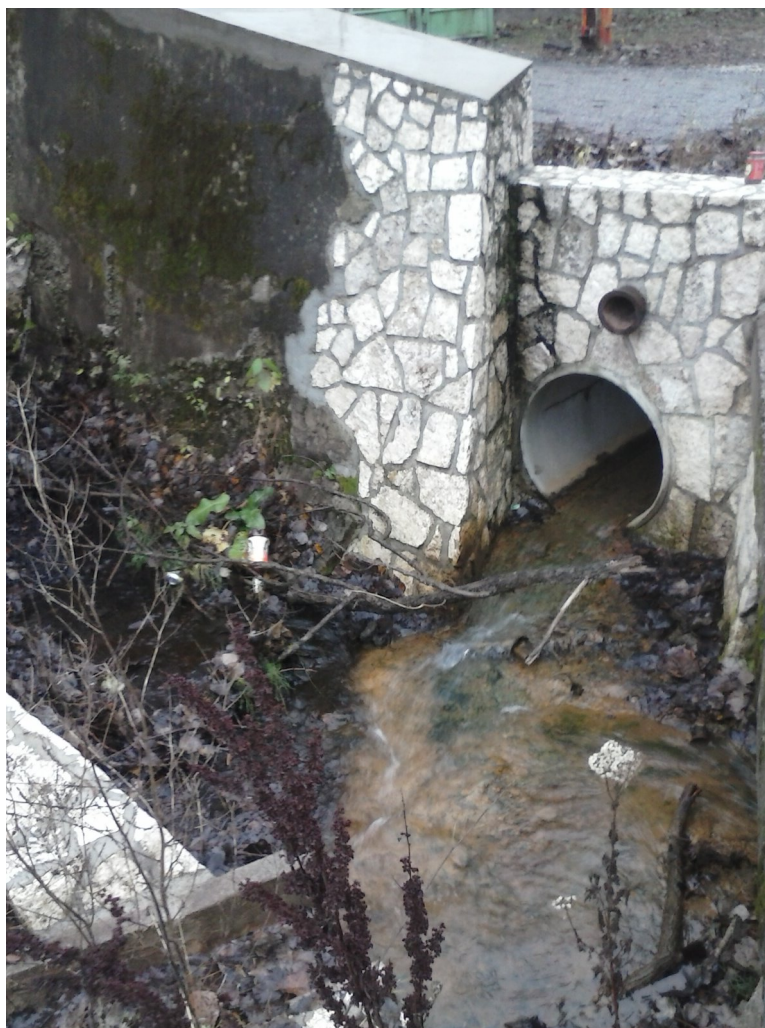

**Fig. S1** Sample site of drainage water flowing out from Elizabeth's shaft in Slovinky (Slovakia)

Supplement: Supplementary file 1 — Supplementary material 1 (PDF 3921 KB) [file 284_2018_1472_MOESM1_ESM.pdf]
