# Supplementary material for: The Bacterial Population of Neutral Mine Drainage Water of Elizabeth’s Shaft (Slovinky, Slovakia)
Source: Curr Microbiol. 2018 Mar 12;75(8):988–96. doi: 10.1007/s00284-018-1472-6 (PMC7160218; doi:10.1007/s00284-018-1472-6)
Supplement: Supplementary file 2 — Supplementary material 2 (PDF 213 KB) [file 284_2018_1472_MOESM2_ESM.pdf]

The bacterial population of neutral mine drainage water of Elizabeth's shaft (Slovinky, Slovakia)

Jana Kisková[1*], Zuzana Perháčová[2], Ladislav Vlčko[2], Jana Kaduková[1], Simona Kvasnová[3], Peter Pristaš[1, 4]

[1]Institute of Biology and Ecology, Faculty of Science, Pavol Jozef Šafárik University in Košice, Košice 041 54, Slovakia

[2]Department of Biology and General Ecology, Faculty of Ecology and Environmental Sciences, Technical University in Zvolen, Zvolen 960 53, Slovakia

[3]Department of Biology and Ecology, Faculty of Natural Science, Matej Bel University, Banská Bystrica 974 01, Slovakia

[4]Institute of Animal Physiology, Slovak Academy of Sciences, Košice 041 01, Slovakia

*Corresponding autor

Email: jana.kiskova@upjs.sk

Table S1 Summary of sequencing effort and number of OTUs detected in microbial community in neutral mine drainage water of Elizabeth‘s shaft (Slovinky, Slovakia)

	**No. of reads, resp. OTUs**
No. of input sequences	9229
After denoising	8910
After trimming	7949
After chimeras and contaminants removing	7095
Assigned to Bacteria	7093
Assigned to Archaea	2
No. of OTUs	813
No. of OTUs represented by single sequence	398
No. of bacterial OTUs	413
No. of archaeal OTUs	1

Table S2 Bacterial groups according to RDP classification [1]

Bacterial group	No. of sequences	No. of OTUs
Known taxonomy up to genus level	6645	329
Known phylum – unknown whole classification	394	75
Candidate phylum	54	9

1. Wang Q, Garrity GM, Tiedje JM, Cole JR (2007) Naïve Bayesian classifier for rapid assignment of rRNA sequences into the new bacterial taxonomy. Appl Environ Microbiol 73(16):5261-5267
